# Patterns of practice for adaptive and real-time radiation therapy (POP-ART RT) part I: Intra-fraction breathing motion management

**DOI:** 10.1016/j.radonc.2020.06.018

**Published:** 2020-12

**Authors:** Gail Anastasi, Jenny Bertholet, Per Poulsen, Toon Roggen, Cristina Garibaldi, Nina Tilly, Jeremy T Booth, Uwe Oelfke, Ben Heijmen, Marianne C Aznar

**Affiliations:** a St. Luke's Cancer Centre, Royal Surrey Foundation Trust, Radiotherapy Physics, Guildford, United Kingdom; b The Institute of Cancer Research and The Royal Marsden NHS Foundation Trust, Joint Department of Physics, London, United Kingdom; c Division of Medical Radiation Physics, Department of Radiation Oncology, Inselspital, Bern University Hospital, Switzerland; d Aarhus University Hospital, Department of Oncology and Danish Center for Particle Therapy, Aarhus, Denmark; e Varian Medical Systems Imaging Laboratory GmbH, Applied Research, Dättwil AG, Switzerland; f European Institute of Oncology IRCCS, IEO-Unit of Radiation Research, Milan, Italy; g Elekta Instruments AB, Stockholm, Sweden; h Medical Radiation Physics, Department of Immunology, Genetics and Pathology, Uppsala University, Sweden; i Royal North Shore Hospital, Northern Sydney Cancer Centre, Australia; j Erasmus MC Cancer Institute, Department of Radiation Oncology, Rotterdam, Netherlands; k Division of Cancer Sciences, Faculty of Biology, Medicine and Health, The University of Manchester, The Christie NHS Foundation Trust, Manchester, United Kingdom; l Nuffield Department of Population Health, University of Oxford, United Kingdom

**Keywords:** Intra-fractional motion, Gating, Tumour tracking, Breath hold, Real-time respiratory motion management

## Abstract

**Purpose:**

The POP-ART RT study aims to determine to what extent and how intra-fractional real-time respiratory motion management (RRMM) and plan adaptation for inter-fractional anatomical changes (ART), are used in clinical practice and to understand barriers to implementation. Here we report on part I: RRMM.

**Material and methods:**

A questionnaire was distributed worldwide to assess current clinical practice, wishes for expansion or new implementation and barriers to implementation.

RRMM was defined as inspiration/expiration gating in free-breathing or breath-hold, or tracking where the target and the beam are continuously realigned.

**Results:**

The questionnaire was completed by 200 centres from 41 countries. RRMM was used by 68% of respondents (‘users’) for a median (range) of 2 (1–6) tumour sites.

Eighty-one percent of users applied inspiration breath-hold in at least one tumour site (breast: 96%). External marker was used to guide RRMM by 61% of users. KV/MV imaging was frequently used for liver and pancreas (with fiducials) and for lung (with or without fiducials). Tracking was mainly performed on robotic linacs with hybrid internal-external monitoring.

For breast and lung, approximately 75% of respondents used or wished to implement RRMM, which was lower for liver (44%) and pancreas (27%). Seventy-one percent of respondents wished to implement RRMM for a new tumour site. Main barriers were human/financial resources and capacity on the machine.

**Conclusion:**

Sixty-eight percent of respondents used RRMM and 71% wished to implement RRMM for a new tumour site. The main barriers to implementation were human/financial resources and capacity on treatment machines.

## Highlights

• Real-time respiratory motion management (RRMM) practice, evaluated for 200 centres.
• Sixty-eight percent of respondents used RRMM for at least one tumour site.
• Across all tumour sites, external marker was the main RRMM signal used.
• Overall 71% of respondents wished to implement RRMM for a new treatment site.
• The main barriers were human/financial resources and capacity on the machine.

It is well documented that tumours in the thorax and abdomen are susceptible to respiratory motion [1], [2], [3], [4]. For “passive” motion management approaches, planning target volumes (PTV) are often defined by encompassing the entire tumour motion observed on a 4DCT (internal target volume (ITV) approach) or by using a statistical margin recipe (e.g. mid-ventilation approach) [5], [6]. These approaches often result in large PTV volumes, which may lead to increased normal tissue toxicity or potentially hamper tumour dose intensification. In contrast, active real-time respiratory motion management (RRMM) approaches (i.e. gating or tracking) may increase targeting accuracy and allow a safe margin reduction and/or dose intensification [3], [7], [8], [9], [10]. Gating involves turning the beam on only when the target is in the desired location while the patient is in free-breathing (FB) or in breath-hold (BH). Tracking involves continuous beam-target realignment. For breast and lung cancer, inspiration BH also results in dosimetrically more favourable lung volume and target-to-heart separation [11]. There is compelling evidence that RRMM improves the delivered dosimetric accuracy [7], [12], [13], [14], [15]. This, combined with some evidence of improved clinical outcome [16], [17], [18], points to RRMM approaching standard of care for specific indications like left breast. The AAPM TG76 report recommends the use of active motion management whenever respiratory motion exceeds an amplitude of 5 mm and/or if it can significantly improve OAR sparing or is needed to achieve clinical goals [19]. This is especially desirable for SBRT where optimal OAR sparing is often required to allow dose intensification.

The use of respiratory gating to improve radiotherapy delivery in the treatment of mobile tumours was first described over 30 years ago [20], [21], but the dissemination of RRMM approaches has long been hampered by the lack of commercially available technology. Today, gating is feasible on the majority of beam delivery systems using breathing surrogates, but imposes a reduced duty cycle in free-breathing while BH requires patient compliance. Tracking is more time-efficient but more technically complex and is currently only commercially available on specialized platforms [22], [23]. MLC tracking on a conventional (C gantry) linac was demonstrated clinically for lung cancer patients in a research setting [24]. Couch tracking may also be used to address respiratory motion but has not been demonstrated clinically to date [25]. Technical challenges for RRMM include handling the software/hardware connectivity such as the fast feedback loop to adapt the beam delivery settings, and also motion monitoring e.g. the uncertainty in correlation between surrogate and target motion, particularly for breathing surrogates [26], [27], [28].

Current commercial RRMM solutions cover a wide range of combinations of monitoring signals and RRMM techniques depending on the available treatment platform, software and add-ons [26]. Intra-fraction motion monitoring, a requirement for the implementation of RRMM, represents a substantial challenge in itself. Other challenges include additional hardware cost, workload and daily treatment time, the need for different QA procedures [29], [30], [31] and appropriate staff and patient training. Though RRMM can be considered standard-of-care in some tumour sites (e.g. deep-inspiration breath-hold in left-sided breast cancer) [32], it is unclear how many institutions have RRMM capabilities and how many patients are treated with RRMM today. Neither is there an overview of experienced hindrances and barriers.

The patterns of practice for adaptive and real-time radiation therapy (POP-ART RT) survey was designed to determine to which extent and how RRMM and Adaptive Radiotherapy (ART) are used in clinical practice in external beam photon RT. In addition, the survey aimed to identify the barriers to implementation or further use to help promote the safe and effective use of these methods as a standard of care. The present paper focuses on the first part of the survey: RRMM. The second part of the survey, focusing on ART for coping with inter-fractional changes taking place on a longer timescale [33] is the topic of an accompanying paper [34].

## Materials and methods

Development of the survey started at the 2nd ESTRO physics workshop topic ‘Real-time and adaptive management of anatomical variation’ (Málaga, October 2018) that gathered clinical, research and industry physicists and one clinical oncologist. The clarity of the questions and completeness of multiple choice answers was improved with the help of three independent physicists (not present at the workshop). The final web-based questionnaire available as supplementary material was distributed and promoted via mailing lists, web articles and social media between February and July 2019 (see acknowledgements). Institutions that were not (yet) using RRMM/ART were explicitly encouraged to also respond and fill the “wish-list and barriers” questions.

Responding centres (“respondents” hereafter) were included in the analysis when they provided a complete response or only isolated questions had not been answered. Where there was no answer or when the authors were unable to interpret the answer, this was designated as “not specified” or “unknown”.

### Analysis and definitions

Responding centres were asked if they were *private*, *public* and/or *academic* centres (with more than one choice possible, question (Q)3, page (p)3) and the number of patients treated with external RT per year (Q4, p3). Respondents were subsequently categorized into *low volume* (<1000 patients/year), *medium volume* (1000–2000 patients/year) and *large volume* (>2000 patients/year) centres. The gross national income per capita (GNI/n) for the year 2018 [35] was used to group respondents into *low*, *middle* and *high* income countries [36] (Q2, p3).

RRMM was defined as the use of gating (FB or BH) or tracking defined as continuously realigning the target and the beam (via robotic, gimbal, MLC or couch tracking) (p4).

Five RRMM techniques were considered (Q4, p7):

1) (deep) Inspiration Breath-hold
2) Expiration Breath-hold
3) Free-breathing inspiration gating
4) Free-breathing expiration gating
5) Tracking

Responding centres using RRMM (‘users’ hereafter), were asked about patterns of practice (patient selection criteria Q1–2, p6 and workflow and technological approaches Q3–8, p7–10) for four main tumour sites, namely breast, lung, liver and pancreas, but were able to specify other sites.

### Wish-lists and barriers to implementation

Users were asked if they wished to increase the use of RRMM or modify their technique in the next two years (p11). Barriers to implementation or further implementation were ranked in order of importance (Q2, p12).

Participants could select a barrier as *not relevant* for their institution by leaving its rank blank. All respondents (users and non-users) were asked if they wished to implement RRMM for any new tumour site (p13) and rank the same barriers to this.

## Results

The RRMM part of the questionnaire was completed by 200 institutions from 41 countries. There were no respondents from the low-income group and only 20 from the middle-income group (Table A.1). Sixty-eight percent (136/200) of all respondents used RRMM for a median (range) of 2 (1–6) tumour sites (Table 1). The most common sites were breast (111/200), lung (89/200), liver (62/200), pancreas (41/200) and lymphoma (14/200). In addition, three users reported using RRMM for ‘mediastinum’, two for ‘heart’,’ oesophagus’ or ‘abdomen’ and one user each for ‘thymoma’, ‘mesothelioma’, ‘adrenal’, ‘stomach’ or ‘suprarenal’ tumours. RRMM was more prevalent in high-income countries than middle-income countries and in academic centres compared to private and public centres (Table A.21).

**Table 1:** Percentage of all respondents (*N* = 200) using gating or tracking to manage respiratory motion for specific treatment sites or overall.

Type of motion management	FB inspiration gating [%]	FB expiration gating [%]	(deep) inspiration BH [%]	Expiration BH [%]	All gating (FB/BH) [%]	Tracking^1^ [%]	Unknown^2^ [%]	All including (excluding) unknown [%]
Breast	1	0	53	<1	**54**	1	1	**56 (55)**
Lung	13	11	17	4	**32**	10	6	**45 (39)**
Liver	6	8	9	8	**22**	8	3	**31 (29)**
Pancreas	4	5	6	6	**15**	5	2	**21 (19)**
Lymphoma	0	0	7	<1	**7**	0	0	**7 (7)**
**Any site**	**13**	**13**	**55**	**10**	**62**	**10**	**7**	**68 (65)**

Abbreviations: BH = breath-hold, FB = free-breathing.

1 One respondent reported using MLC tracking in a trial, all other users used CyberKnife.

2 For respondents reporting to do tracking on conventional linacs (without further explanation about the use of commercially unavailable technology), the authors assumed that tumour motion was monitored but that the beam was not realigned with the target.

**Fig. 1:**
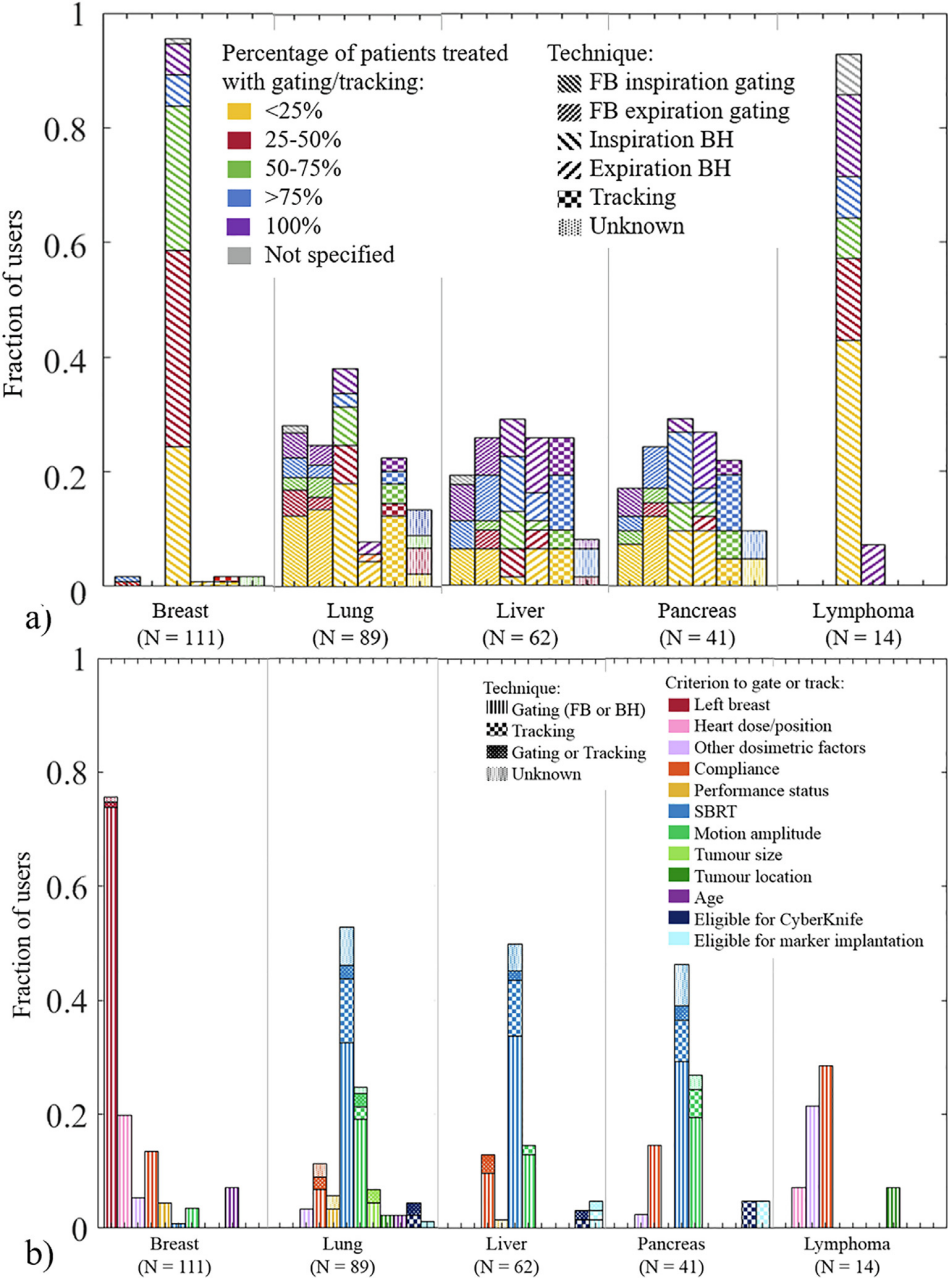
a) Fraction of users that use a given technique. The pattern of each column indicates the technique, The colour of each column segment indicates the percentage of patients receiving gated/tracked treatment. Respondents could use more than one technique per treatment site. A substantial number of respondents reported using tracking but it was unclear if tracking was meant as monitoring only. b) Fraction of users that use given selection criteria to decide to treat patients with gating or tracking. Respondents could use more than one criterion per treatment site.

Within any given tumour site, users generally only applied RRMM in selected patients (Fig. 1a). Most users applied RRMM for <25% of lung, pancreas and lymphoma patients, for 25–50% of the breast patients and for >75% of the liver patients. Five users who indicated using RRMM for 100% of patients, commented that it was 100% of stereotactic body radiotherapy (SBRT) patients.

The main selection criteria reported for breast patients were ‘left breast’ (76%) and ‘heart dose/position’ (20%), while for lung, liver and pancreas, the main criteria were ‘SBRT’ (~50%) followed by ‘tumour motion amplitude’ (Fig. 1b).

Inspiration BH was the dominant technique among RRMM users for breast (96%), lymphoma (93%) and lung (38%) (Fig. 1a). Gating was performed on linacs, except for one user employing Tomotherapy (Accuray Inc, Sunnyvale, CA) for expiration gating (lung) and four users performing gating on an MR-linac. Fifteen percent of users were using tracking (Fig. 1a) with a higher prevalence in private and academic centres than public ones (Table A.2). Tracking is currently only commercially available on the Cyberknife (Accuray) or Vero (BrainLab and Mitsubishi Heavy Industries, Japan) platforms. Cyberknife was used by all tracking users except one which used a conventional linac as part of a clinical trial for electromagnetic-guided MLC tracking (lung) [24]. For all other respondents who reported tracking on conventional linacs, we assumed they were monitoring tumour motion but not actually realigning the target and the beam in real-time. Their RRMM technique was designated as “unknown”. No Vero user responded to the survey. No centre from middle-income countries reported tracking (Table A.2, Fig. A.1). In the following, users who responded doing both gating and tracking for a given treatment site are considered as a separate group from gating only or tracking only because it was not possible to determine to which technique following responses applied.

**Fig. 2:**
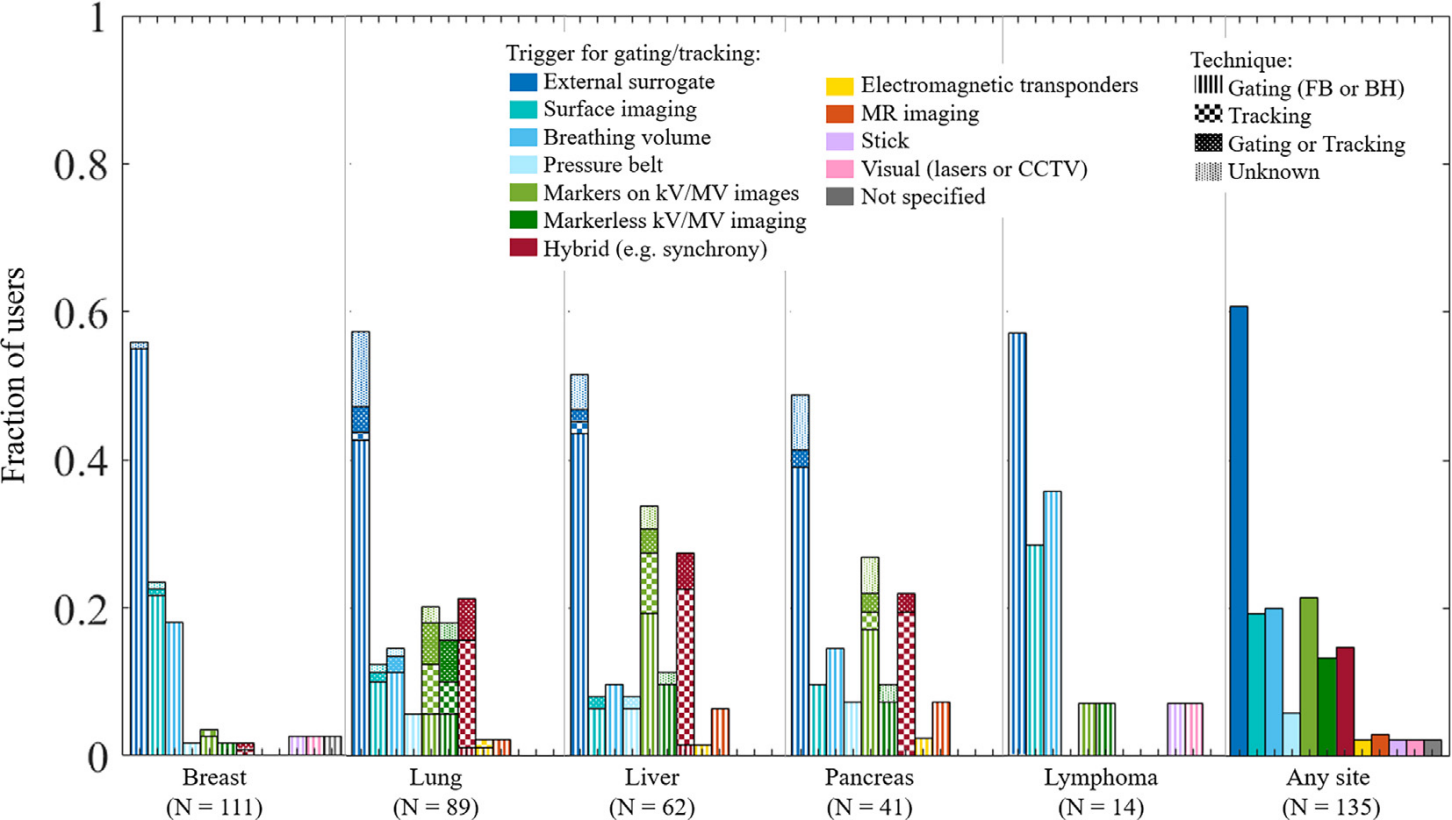
Fraction of users that use a given signal to trigger the gating or control the tracking feedback loop (alone or in combination).

Across all tumour sites and techniques, external marker (e.g. RPM) was the main RRMM signal, used by 61% of users (Fig. 2). For breast, surface imaging was used by 23% of users. KV/MV imaging was frequently used for liver and pancreas (with fiducials) and for lung (with or without fiducials). A hybrid RRMM technique was used by all Cyberknife users (Synchrony) [15] and by one linac user for gating with the BrainLab beam delivery system [37]. No user from a middle-income country reported use of MR, surface or electromagnetic guidance for RRMM (Fig. A.2).

Under half of the users who employed surface imaging or a breathing surrogate (external marker, breathing volume, pressure belt), acquired verification images during beam-on (Table A.3). However when acquired, verification images were generally looked at online.

**Fig. 3:**
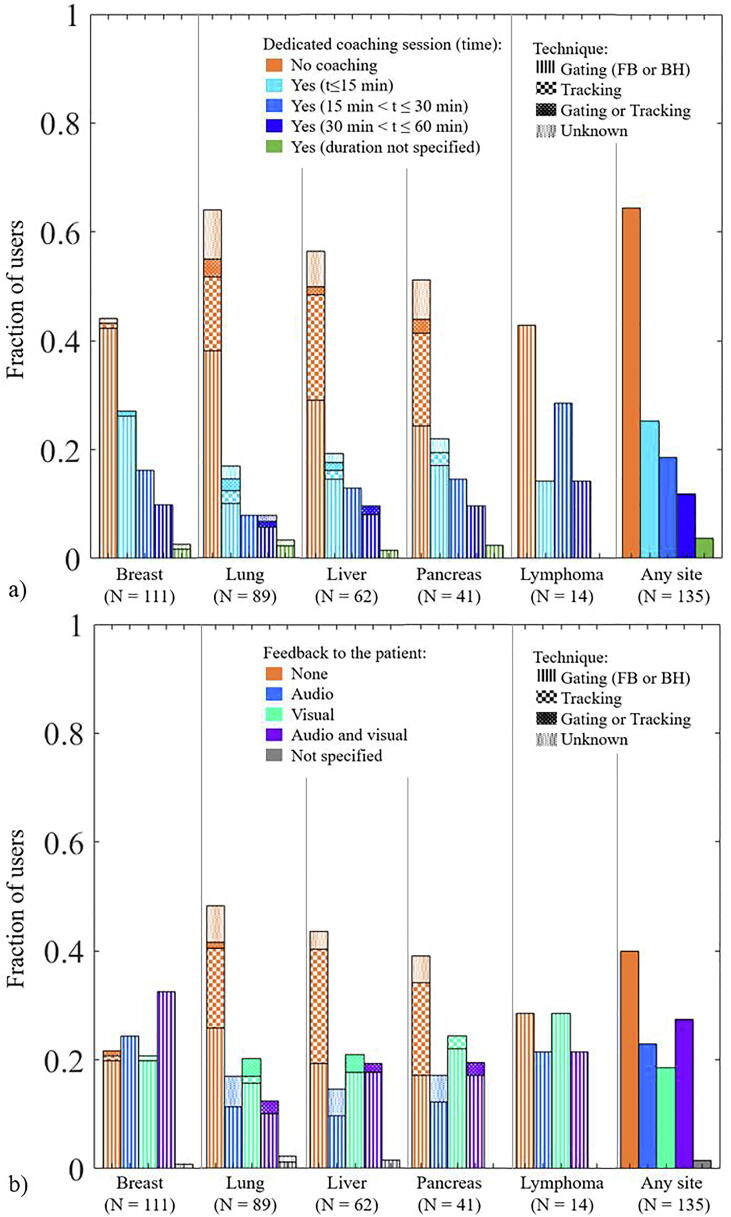
a) Fraction of users using gating or tracking that use a separate coaching session. b) Fraction of users doing gating or tracking that use audio and/or visual feedback to the patient.

A dedicated coaching session was used by over half of the users treating breast and lymphoma with gating (mostly <15mins, Fig. 3a). Audio and/or visual feedback was used by >70% of users for lymphoma and breast and by just above 50% of users for lung, liver and pancreas (Fig. 3b). Coaching and feedback were generally not used in combination with tracking (Fig. 3).

**Fig. 4:**
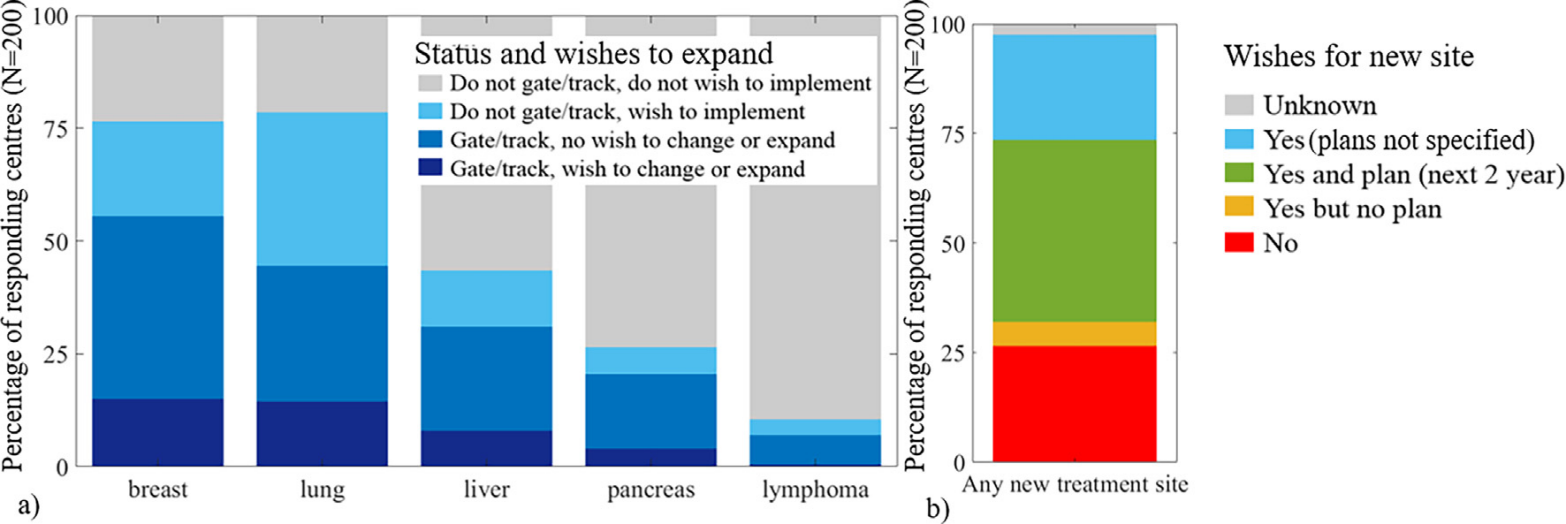
a) Percentage of respondents using gating/tracking with (dark blue) and without (medium blue) a wish to change technique or increase the number of patients having gating/tracking, respondents not applying gating/tracking with (light blue) and without (grey) a wish to implement it. b) Overall percentage of respondents wishing to implement gating or tracking for any new treatment site (blue, green and yellow) or not (red). (For interpretation of the references to colour in this figure legend, the reader is referred to the web version of this article.)

For breast and lung, 36% and 49% of respondents respectively wished to expand/change their technique or implement RRMM (Fig. 4a). For liver and pancreas >55% of respondents did not use RRMM and did not wish to implement it in priority, in contrast to <25% for breast and lung. Overall 71% of respondents wished to implement RRMM for a new treatment site (Fig. 4b). In addition to the tumour sites mentioned in the survey, nine respondents wished to implement RRMM for abdominal sites and five for oesophagus.

**Fig. 5:**
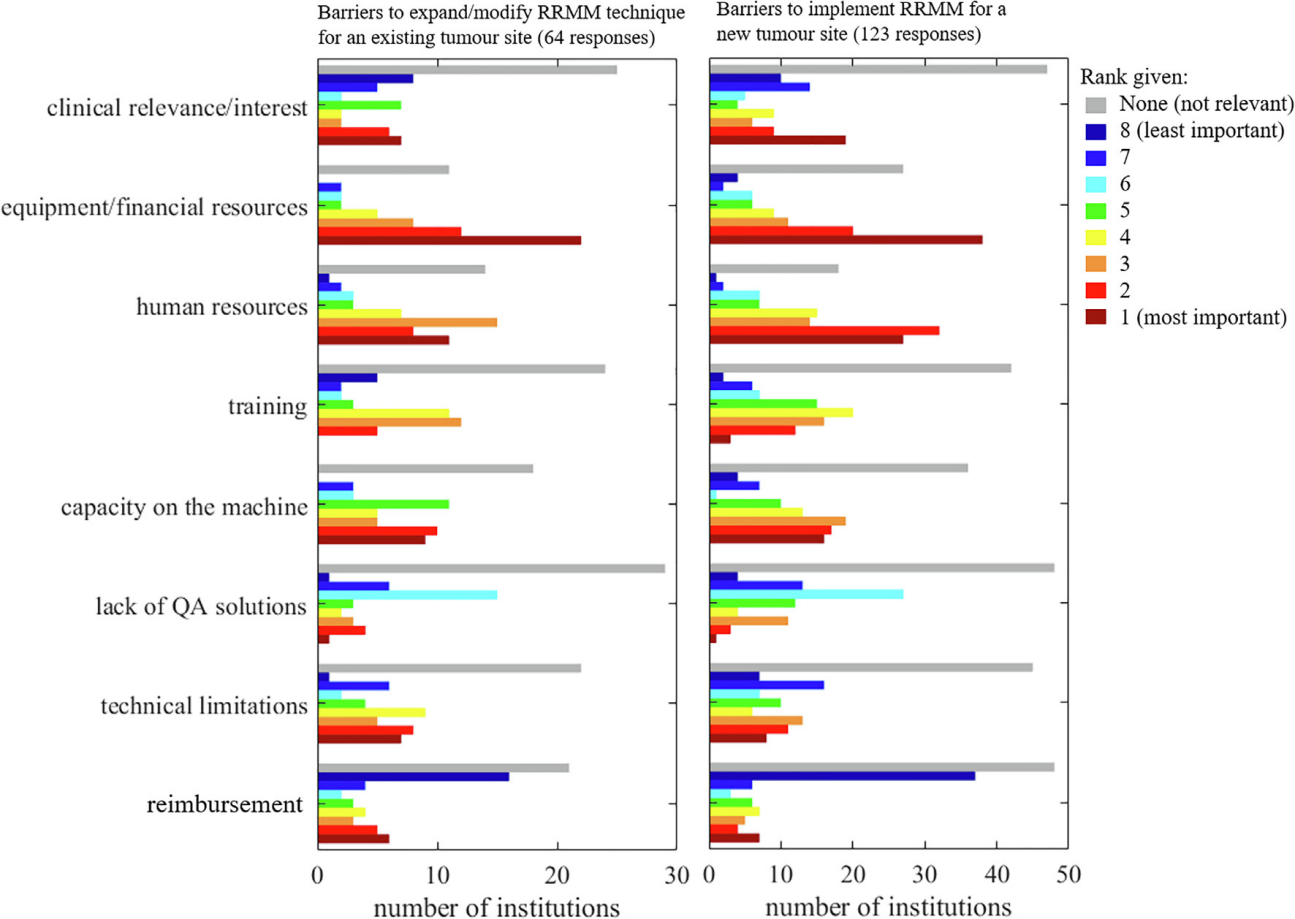
Histogram of the barriers to further use for an existing RRMM tumour site (left) or implementation for a new tumour site (right). Colour indicating increase in importance from blue colour (low) towards red colour (high). The grey bars indicate the number of institutions that considered the barrier “not relevant”. (For interpretation of the references to colour in this figure legend, the reader is referred to the web version of this article.)

Sixty-four users ranked the barriers to further use of RRMM. *Equipment/financial resources* was ranked first or second by 34 respondents (first to third by all middle-income countries). *Human resources* and *capacity on the machine* were also considered highly important by a majority of respondents. Although most respondents rated *reimbursement* as not relevant or having a low importance, 8% of respondents still rated it as the main limitation (Fig. 5).

The barriers to implementing RRMM for new tumour sites were ranked by 123 respondents. *Human resources* was almost equally important as *equipment/financial resources* followed by *capacity on the machine*. *Reimbursement* remained lowly ranked.

Barriers entered as *other* and comments on the barriers included ‘limited linacs with necessary equipment’ or ‘waiting for MR-linac’ (four respondents), ‘increased time for treatments’ (two respondents), ‘lack of time to develop/implement new techniques’ (two respondentss), ‘multi-disciplinary cooperation’ (two respondents), ‘patient compliance’ (two respondents), ‘lack of national target’ (one respondents), ‘approval from authorities’ (one respondent).

The ranking of barriers did not differ substantially from the overall ranking when analysed by type of institution or socio-economic status although the number of responses was occasionally very small (Fig. A.3).

## Discussion

This study reports on the patterns of practice for RRMM in 200 RT centres from 41 countries worldwide.

Sixty-eight percent of respondents used RRMM for at least one tumour site (Table 1), with a median (range) number of tumour sites per user of 2 (1–6). Eighty-one percent of RRMM users applied inspiration BH in at least one tumour site.

Despite our explicit definition of tracking as active realignment of the beam and the moving target, there was confusion among some respondents who indicated doing tracking on conventional linacs. Since this option is not commercially available, despite active research in the past decades [38], [39], [25], [40], we attempted to contact those respondents who confirmed that they were only monitoring motion (visual tracking, as opposed to active beam/target re-alignment). When the correct answer could not be confirmed (7 users), we indicated ‘unknown’ as the RRMM technique. It was confirmed that tracking was used in 10% of respondents while gating (BH or FB) was used by 62%.

The proportion of patients being offered RRMM varied according to tumour site. For example, where RRMM was employed to treat liver tumours, it tended to be offered to a large proportion of patients (mode: >75%, Fig. 1a). In contrast, RRMM was mostly used for 25–50% of breast cancer patients and <25% of lung cancer patients (Fig. 1a). One explanation could be that, given a relatively small volume of liver patients, the workload remains manageable, while the larger patient volume for breast and lung necessitates stricter patient selection.

At the time of data collection some clear patterns of practice were highlighted in this fast evolving field. Inspiration BH was the dominant RRMM technique for breast and lymphoma, whereas the spread in technique was greater for other sites (Fig. 1a). The reported selection criteria reflect the clinical evidence of heart-sparing in left-breast (Deep) Inspiration BH [16] and the need for higher targeting accuracy in SBRT [12], [13]. Note also that for lung, liver and pancreas, some users that indicated treating 100% of patients with RRMM specified that it was SBRT patients only. For lung cancer, SBRT is often used for small mobile tumours, while, for locally advanced lung cancer, the dosimetric impact of intrafractional motion (including respiration) is often smaller than that of large interfractional anatomical changes, which are addressed with ART [34].

Across all tumour sites, an external marker surrogate was the main RRMM signal used by 61% of users (Fig. 2). While kV/MV imaging was often reported, it was mostly in combination with a breathing surrogate. It remains unclear if image-based monitoring was performed automatically or as visual verification for the breathing surrogate. To our knowledge, there is only one commercial solution available for gating on conventional linacs that combines automatic fiducial monitoring on kV images with external marker monitoring [41].

Less than half of users employing a breathing surrogate acquired verification images during treatment. However, there is evidence that residual errors between breathing surrogates and internal target motion may be substantial [42], [43], [44], [45]. This reflects the lack of practical intra-fraction monitoring solutions for internal targets and the need to integrate such solutions into the clinical workflow. Daily pre-treatment assessment and correction of the mean tumour position, required for non-breast tumours [46], was not covered by the questionnaire.

Hybrid monitoring, where the external-internal correlation is explicitly considered and verified during delivery, was used for Cyberknife-based tracking, with markerless tumour motion monitoring for certain lung tumours [17].

Over 75% of respondents wished to implement or change/increase their use of RRMM for lung and breast in priority whereas this was the case for only <50% of the respondents for liver and pancreas. Note that some of these respondents might not offer liver or pancreas RT at all. Respiratory motion amplitude is often larger in the abdomen compared to the thorax [1], [2], [3], [4] but motion monitoring is also more challenging due to poor soft tissue visualization on kV/MV imaging. MR-linacs provide better soft tissue contrast, facilitating RRMM in the abdomen, provided that motion mitigation is available [47], [48].

Over 40% of respondents had plans to implement RRMM for a new treatment site within the next two years (Fig. 4b), meaning a significant rise in RRMM can be expected. Twenty-four percent of the respondents that already used RRMM for at least one treatment site had no wish to implement RRMM for a new treatment site. Only 3% of respondents were not users and had no wish to implement RRMM for any site. The main barriers of human/material resources are most likely due to the need for additional equipment which comes at significant cost and an increased need in staff to cover different platforms/equipment. Although this survey did not cover RRMM commissioning and QA in detail, their importance and associated added workload cannot be underestimated, especially for centres implementing RRMM for the first time. Hardware QA was documented by De Los Santos et al [29], while treatment-delivery QA such as automated 4D dose reconstruction has been demonstrated clinically (in real-time or offline) in research settings [8], [49]. Full verification of RRMM requires discretization of treatment delivery into small time-increments. This time-resolved evaluation process represents a paradigm shift in treatment verification. In contrast, verification for ART [34] can be performed on a per-fraction basis (e.g. using log-files, secondary dose calculation) where the delivered dose at each fraction can be evaluated in a similar manner as full-course plans.

Of the 200 centres who completed the RRMM part of the questionnaire, 177 centres also completed the part covering ART [34]. Offline replanning, where plan verification can be performed essentially in the same way as for non-ART cases, was applied by 50% of respondents. We encourage the reader to see the accompanying paper for expansion of common results and discussions [34].

With only twenty respondents from middle-income countries, it is difficult to draw conclusions based on socio-economic status. The human/material resources needed for ART/RRMM are expected to be less available in middle-income countries [50], [51] which may explain why no respondent from middle-income countries used tracking or MR, surface or electromagnetic guidance to trigger gating. For ART [34], no respondents from middle-income countries was using daily online replanning which is also the most demanding in terms human/material resources.

A limitation for both parts of this study is the bias in the representation of respondents. Most respondents were public or academic centres in high-income countries. Centres doing or having an interest in RRMM/ART may have been more likely to respond, despite our encouragement to non-users to respond. This bias may have had a particularly strong impact for centres from middle-income countries. In addition, the survey was only available in English and was promoted and completed on the internet which may have resulted in a low number of responses from countries where English is not a commonly spoken language or where internet access is low. Other limitations include a) the subjectivity of the respondent for the wish-lists and barriers questions which may represent their personal assessment rather than the consensus opinion of the centre b) the survey was mostly addressed to physicists. Hospital administrators might have other views of the barriers. Nonetheless, we believe that with 135 users (108 for ART [34]), this study gives an interesting insight into how RRMM and ART are used currently as well as the wishes for expansion/changes. In addition with 65 non-users (69 for ART), the study provides important information on barriers to implementation

Based on our results, the required next steps to promote the safe and effective use of RRMM as a standard of care are:

1) that manufacturers provide practical, low-cost internal verification monitoring solutions for internal targets on conventional linacs, particularly where an external breathing surrogate is used.
2) that such solutions be integrated into the clinical workflow with minimal increase in treatment time and workload.
3) that research studies providing evidence of improvement in clinical outcomes as direct result of RRMM are performed to support clinical relevance/interest.

In conclusion, 68% of respondents used RRMM for at least one tumour site, primarily with gating (in free breathing or in breath-hold) using external marker. Although RRMM was common in the thorax, it was generally applied for less than half of the patients. Further, within the same tumour site, there is a large disparity among respondents with regards to the number of patients selected for RRMM. There is an unmet need for RRMM, particularly in lung cancer where 49% of respondents wished to expand or implement RRMM. More than 40% of the respondents have plans to implement RRMM within two years but the main barriers were human/material resources and machine capacity.

To further promote safe and effective use of both ART and RRMM and to reduce the strain on human/material resources, we recommend that users, future users and vendors work together towards efficient solutions and workflows available for use on conventional equipment. Further, consensus on best practice is needed for the establishment of clear, broadly accepted guidelines. This could also contribute to development of solid and consistent reimbursement practices.

## Conflicts of interest

Jenny Bertholet and Uwe Oelfke declare that the ICR is part of the Elekta MR-linac Research consortium.

Toon Roggen declares that he is an employee of Varian Medical Systems.

Nina Tilly declares that she is an employee of Elekta Instruments AB.

Other co-authors have no conflict of interest to declare in relation to the present work.

## Funding section

This paper is part of twin publication. Similar to part II (https://doi.org/10.1016/j.radonc.2020.06.017).

## Figures and Tables

**Supplementary figure 1:**
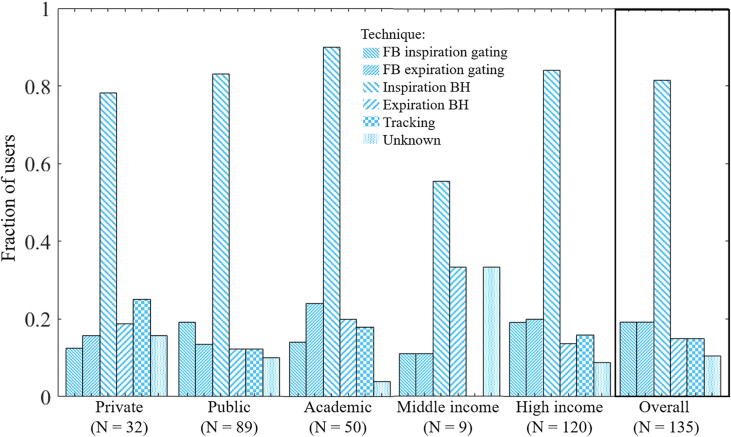


**Supplementary figure 2:**
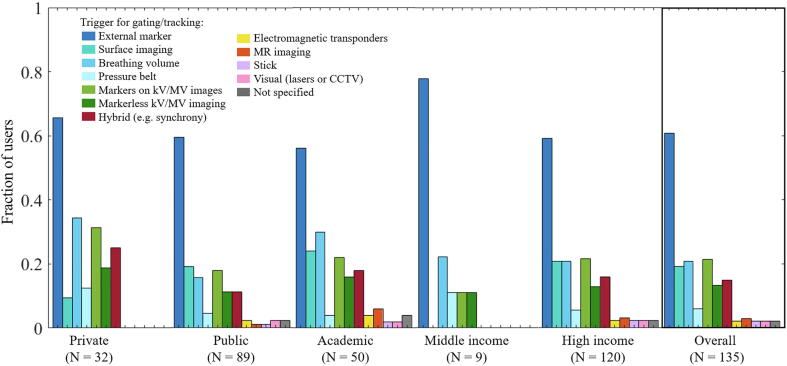


**Supplementary figure 3:**
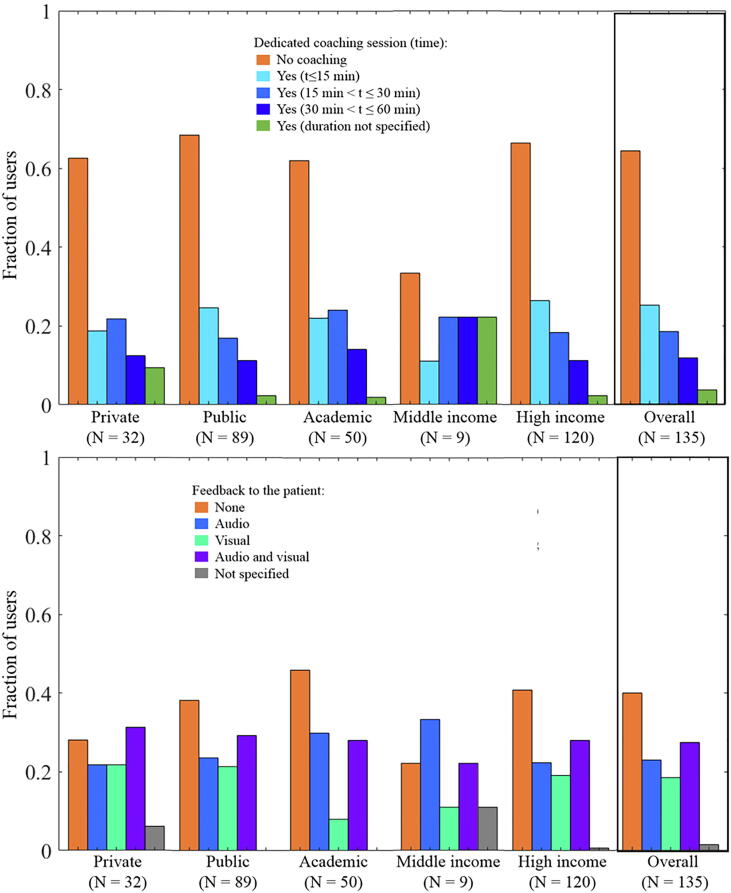


**Supplementary figure 4:**
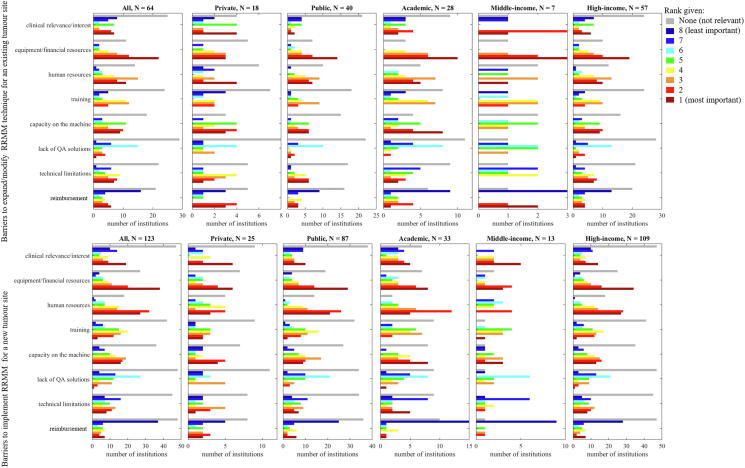


## Appendix A Supplementary data

Supplementary data: web-based questionnaire
