# Supplementary material for: Patterns of practice for adaptive and real-time radiation therapy (POP-ART RT) part I: Intra-fraction breathing motion management
Source: Radiother Oncol. 2020 Dec;153:79–87. doi: 10.1016/j.radonc.2020.06.018 (PMC7758783; doi:10.1016/j.radonc.2020.06.018)
Supplement: Supplementary Table A.1 [file mmc2.docx]

| Table A.1: Characteristics of respondents. Number (percentage) | | | | | | | |
| --- | --- | --- | --- | --- | --- | --- | --- |
| Group | Overall N = 200 | Type of institution^1^ | | | Economic status^2^ | | |
|  |  | Private  N = 52 | Public  N = 132 | Academic  N = 57 | | Middle-income  N = 20 | High-income  N = 178 |
| Small volume^3^ | 61 (31%) | 25 (48%) | 35 (27%) | 5 (9%) | | 9 (45%) | 50 (28%) |
| Medium volume^3^ | 68 (34%) | 14 (27%) | 46 (40%) | 14 (25%) | | 6 (60%) | 62 (35%) |
| Large volume^3^ | 69 (35%) | 12 (23%) | 51 (39%) | 37 (65%) | | 4 (20%) | 65 (37%) |
| ^1^ Institution could specify more than one type.  ^2^ Information unavailable for two institutions. The “middle-income” group included institutions from Egypt, Indonesia, India, the Philippines, Brazil, Ecuador, Mexico, Peru, Romania, Serbia, Russia and South Africa. The “high-income” group included institutions from Austria, Belgium, Switzerland, Czech republic, Germany, Denmark, Estonia, Spain, Finland, France, Great Britain, Croatia, Hungary, Ireland, Italy, the Netherlands, Norway, Poland, Portugal, Sweden, Slovenia , USA, Canada, Australia, Israel, Hong Kong and the Arab Emirates  ^3^ Number of respondents with small (<1000 patients/year) / medium (1000-2000 patients/year) / large (>2000 patients/year) patient volume (external beam RT only). Two institutions did not specify the patient volume (numbers do not always add up to ‘N’). | | | | | | | |
