# Supplementary material for: Patterns of practice for adaptive and real-time radiation therapy (POP-ART RT) part I: Intra-fraction breathing motion management
Source: Radiother Oncol. 2020 Dec;153:79–87. doi: 10.1016/j.radonc.2020.06.018 (PMC7758783; doi:10.1016/j.radonc.2020.06.018)
Supplement: Supplementary Table A.2 [file mmc3.docx]

| Table A.2: Percentages of all respondents that apply RRMM; overall and according to type of institution and economic status. | | | | | | | | | | | | |
| --- | --- | --- | --- | --- | --- | --- | --- | --- | --- | --- | --- | --- |
| **Group** | **Overall (N=200)** | | **Type of institution^3^** | | | | | | **Economic Status^4^** | | | |
|  |  |  | **Private**  **N = 52** | | **Public**  **N= 132** | | **Academic**  **N=57** | | **Middle-income N=20** | | **High-income N=178** | |
| Type of motion management | **Tracking^1^**  **[%]** | **All^2^ (unknown) [%]** | **Tracking^1^**  **[%]** | **All^2^ (unknown)**  **[%]** | **Tracking^1^**  **[%]** | **All^2^ (unknown) [%]** | **Tracking^1^**  **[%]** | **All^2^ (unknown) [%]** | **Tracking^1^**  **[%]** | **All^2^ (unknown) [%]** | **Tracking^1^**  **[%]** | **All^2^ (unknown)**  **[%]** |
| Breast | 1 | 56 (1) | 0 | 48 (2) | <1 | 58 (<1) | 2 | 74 (0) | 0 | 30 (5) | 1 | 59 (<1) |
| Lung | 10 | 45 (5) | 15 | 50 (10) | 8 | 40 (5) | 17 | 63 (4) | 0 | 35 (15) | 11 | 46 (5) |
| Liver | 8 | 31 (3) | 12 | 35 (6) | 7 | 25 (2) | 13 | 54 (2) | 0 | 30 (5) | 9 | 31 (2) |
| Pancreas | 5 | 21 (2) | 8 | 23 (4) | 3 | 17 (2) | 10 | 42 (2) | 0 | 20 (0) | 5 | 21 (2) |
| Lymphoma | 0 | 7 (0) | 0 | 4 (0) | 0 | 8 (0) | 0 | 15 (0) | 0 | 0 | 0 | 8 (0) |
| **Any site** | 10 | 68 (7) | 15 | 62 (10) | 8 | 68 (7) | 17 | 88 (4) | 0 | 45 (15) | 11 | 70 (6) |
| ^1^Tracking is for all respondents that had tracking in their reply (tracking only or tracking and gating).  ^2^ All is for all respondents that had tracking only, tracking and gating or gating only in their reply.  ^3^Institution could specify more than one type.  ^4^ Information unavailable for two institutions. See Table A.1. | | | | | | | | | | | | |
