# Supplementary material for: Patterns of practice for adaptive and real-time radiation therapy (POP-ART RT) part I: Intra-fraction breathing motion management
Source: Radiother Oncol. 2020 Dec;153:79–87. doi: 10.1016/j.radonc.2020.06.018 (PMC7758783; doi:10.1016/j.radonc.2020.06.018)
Supplement: Supplementary Table A.3 [file mmc4.docx]

| Table A.3: Number of users who take a verification image during treatment when using a breathing surrogate. | | | | | |
| --- | --- | --- | --- | --- | --- |
| Tumour site | N (breathing surrogate) | Do you take verification imaging during beam-on?  N (%) | | | |
|  |  | Yes, we look at them online | Yes, we look at them offline | No | Not specified |
| Breast | 106 | 27 (25%) | 17 (16%) | 56 (53%) | 6 |
| Lung | 72 | 28 (39%) | 6 (8%) | 37 (51%) | 1 |
| Liver | 44 | 17 (39%) | 2 (5%) | 24 (55%) | 1 |
| Pancreas | 31 | 14 (45%) | 1 (3%) | 15 (48%) | 1 |
| Lymphoma | 14 | 2 (14%) | 4 (28%) | 8 (57%) | 0 |
